# A study of the subdiffusion of small molecules in charged polyelectrolyte multilayers

**DOI:** 10.1038/s41598-021-01935-7

**Published:** 2021-11-19

**Authors:** I. Vardanyan, V. Arakelyan, Z. Navoyan, Eleftheria Diamanti, S. E. Moya, E. Donath

**Affiliations:** 1 Department of Molecular Physics, Yerevan State University, 1 Al. Manoogian Str., 0025 Yerevan, Armenia; 2 Soft Matter Nanotechnology Lab, CIC biomaGUNE, 20009 San Sebastian, Spain; 3 Institute of Medical and Biophysics, University of Leipzig, Haertelstrasse 16-18, Leipzig, Germany

**Keywords:** Physical chemistry, Soft materials, Applied mathematics

## Abstract

A theoretical approach has been developed here to describe the slow diffusion of small charged molecules of sodium dithionite (S_2_O_4_^2−^) in polyelectrolyte multilayers (PEMs) composed of polyallylamine hydrochloride (PAH) and polystyrene sulfonate (PSS), which is demonstrated here to be a case of subdifussion. Diffusion is measured experimentally by recording the quenching of the fluorescence of (7-nitrobenz-2-oxa-1,3-diazol-4yl) amino (NBD) labelled PAH layers assembled on silica particles by flow cytometry. NBD is reduced when it encounters dithionite leading to the disappearance of the fluorescence. The fluorescence decay curves show a slow diffusion of dithionite, that does not follow classical Fickean law. Dithionite diffusion in the PEMs is shown to be a non-Markovian process and the slow diffusion can be described via diffusion equations with fractional time derivatives. Results are explained assuming subdifussion of dithionite in the PEMs, as a result of the trapping of the negatively charged dithionite in the positively charged layers of PAH.

## Introduction

The layer-by-layer technique (LbL), based on the sequential assembly of oppositely charged polyelectrolytes is a powerful tool for non-covalent surface functionalisation as well as for the development of hybrid and multifunctional films, so called polyelectrolyte multilayers (PEMs)^1,2^. PEMs feature in various applications ranging from optics, energy, protection, filtration, to biomedicine and drug delivery^2,3^. PEMs are a special case of polyelectrolyte complexation^4^ where layers of oppositely charged polyelectrolytes are assembled both on planar surfaces and colloidal particles^5–7^.

An important property of PEMs in regard to several applications is their permeability^8^. Controlling the diffusion of molecules through PEMs is fundamental for drug delivery applications, and also for the use of PEMs as protective barriers, or in nanofiltration or reverse osmosis applications^9,10^.

The unique architecture of PEMs, consisting of a layered structure where alternating layers bear opposite charges, makes them a fascinating system to study the diffusion of charged molecules. These would be facing alternating attractive and repulsive interactions while diffusing through the film.

In a previous paper we have shown a slow diffusion of the negatively charged small molecule dithionite through multilayers of poly allyl amine hydrochloride (PAH) and poly styrene sulfonate (PSS)^11^. Diffusion of dithionite was followed by a quenching assay using flow cytometry. Dithionite was used to quench the fluorescence of the nitrobenzoxadiazole (NBD) dye molecules, which were covalently linked to PAH chains assembled on top of colloidal particles. The changes in fluorescence of the colloidal particles were followed by flow cytometry. We demonstrated that dithionite diffusion in PEMs does not follow an exponential law and can be considered as atypical.

In this work, we aim to advance on our understanding of the diffusion of charged small molecules in PEMs, and we propose a different theoretical framework for analysing experimental results based on the assumption that diffusion is non-Markovian and the slow diffusion can be described via diffusion equations with fractional time derivatives^13–16^. We develop a mathematical model, which perfectly fits experimental results, and proves subdiffusion of dithionite in the multilayers. The theoretical proof of subdiffusion allows us to propose a mechanism of how dithionite or any other small charged molecule diffuses inside PEMs. This mechanism is based on the fact that during diffusion through the PEM, the negatively charged dithionite interacts with the positively charged amine groups of the film and as a result, the dithionite remains for some time near the positively charged groups, as if being trapped before continuing to diffuse further, which results in a subdifussion process.

## Materials and methods

Here we follow the same procedures as we have done in^11,12^. Silica particles (diam: 3.03 ± 0.17 µm) were purchased from microParticles GmbH (Berlin, Germany). PAH (MW 70,000), PSS (MW 70,000), and Sephadex G-25 chromatography gel were obtained from Sigma (Deisenhofen, Germany). Succinimidyl 6-(N(7-nitrobenz-2-oxa-l,3-diazol-4yl)amino) hexanoate (S-NBD) was purchased from Molecular Probes (Eugene, Oregon). Molecular porous membrane tubing (MWCO: 12,000–14,000 Da) was purchased from Spectrum Laboratories, Inc. (California). All other chemicals were obtained from Fluka (Neu-Ulm, Germany).

### Labelling of PAH

Labelling PAH with S-NBD was performed as in^11^. S-NBD was first dissolved in carbonate buffer, 10% DMF (v/v), and then added dropwise to a solution of PAH dissolved in 0.1 M carbonate buffer at pH 8.5. The mixture was left under stirring for 48 h. Then, the solution was passed through a Sephadex Gel chromatography column to separate the labelled polymer from the free dye. The remaining buffer was removed by dialysis. The labelled and purified polyelectrolytes were lyophilized and stored at 4 °C for future use. UV–Vis measurements to determine the labelling degree were performed on a Cary 50 spectrometer (Varian Instruments) using the molar extinction coefficient ε_484_ = 41,200 M^−1^ cm^−1^ for NBD. Furthermore, labelled PAH will be referred to as PAH^NBD^.

### Particle coating

Silica particles were coated with a PEM as presented in ^11^. Silica particles (500 µL, 5 wt%, approximate surface area 0.025 m^2^) were centrifuged and the product of centrifugation dispersed in 200 µL of 0.5 M NaCl. This suspension was pipetted into a 1 mg/mL solution of PAH in 0.5 M NaCl and incubated under constant stirring for 10 min. Then, the particles were centrifuged at 650×*g* for 2 min and washed 3 times with 0.1 M aiming to remove excess of polyelectrolyte. This was followed by the same procedure to assembly a layer of PSS. The particles were dispersed in a 1 mg/mL solution of PSS in 0.5 M NaCl wa. After PSS adsorption, washing was performed in distilled water. The whole procedure was repeated until 8 polyelectrolyte layers ([PAH-PSS]_4_) were assembled on the top of silica. Then, a layer of PAH^NBD^ was adsorbed followed by an additional PSS layer PAH^NBD^ and PSS were alternatively assembled till reaching 16 layers on top of the particles. In the current work we have used the experimental data obtained for PEMs with the following layers [PAH-PSS]_4_-[PAH^NBD^-PSS]_3_-PAH^NBD^, [PAH-PSS]_4_-[PAH^NBD^-PSS]_4_, [PAH-PSS]_4_-[PAH^NBD^-PSS]_7_-PAH^NBD^, [PAH-PSS]_4_-[PAH^NBD^-PSS]_8_. The assembly of the PAH^NBD^ on the colloids was followed by flow cytometry looking at the increase in fluorescence intensity after each layer deposition.

### Flow cytometry

**Figure 1:**
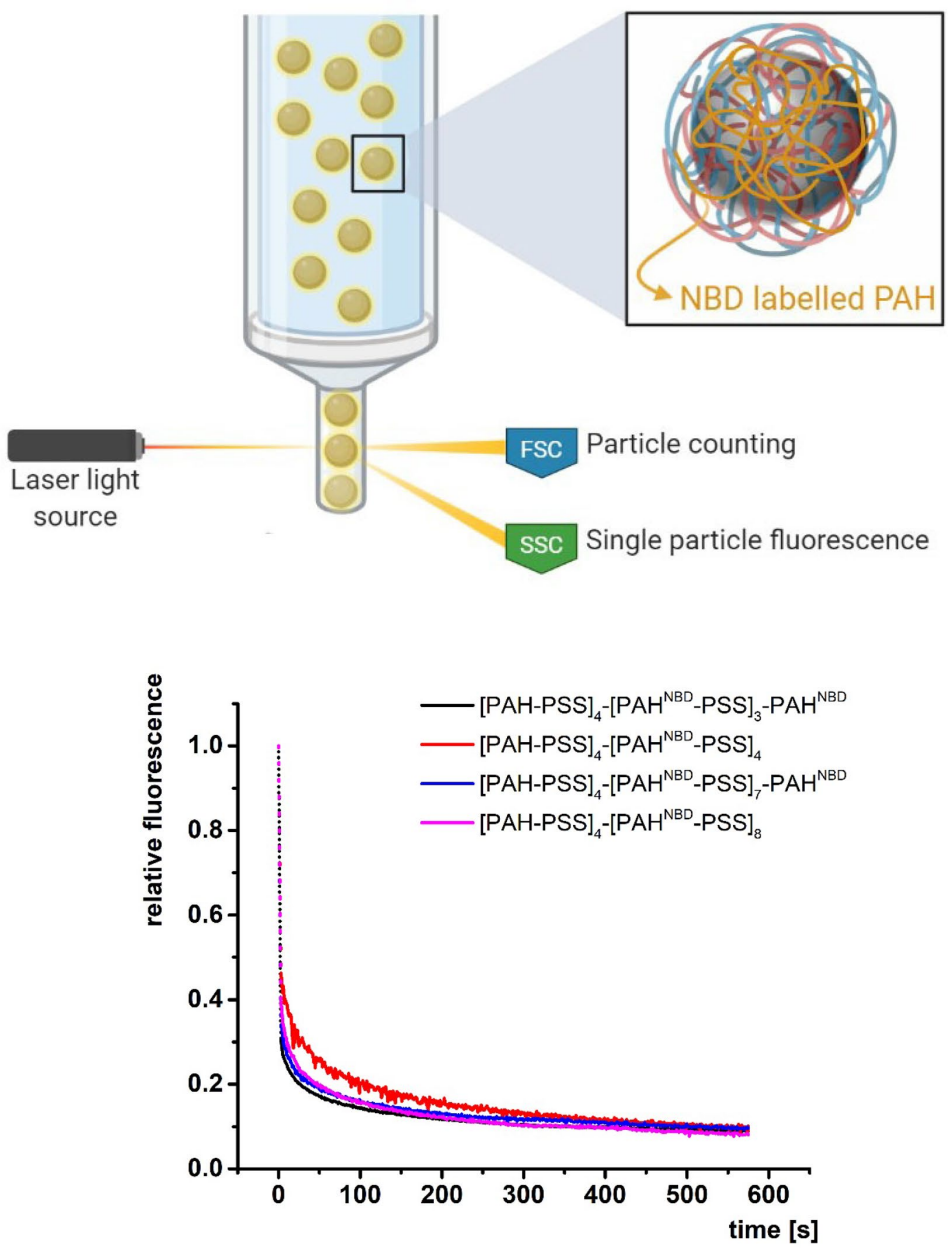
Top, scheme of the methodology for tracing the fluorescence intensity per particle after quenching of NBD with dithionite by flow cytometry. Bottom, the changes in relative fluorescence with respect to time for the PEMs [PAH-PSS]_4_-[PAH^NBD^-PSS]_3_-PAH^NBD^, [PAH-PSS]_4_-[PAH^NBD^-PSS]_4_, [PAH-PSS]_4_-[PAH^NBD^-PSS]_7_-PAH^NBD^, [PAH-PSS]_4_-[PAH^NBD^-PSS]_8_ after adding dithionite. Data have been previously shown in^11^.

Flow cytometry measurements were performed with a FACS Calibur flow cytometer (Becton Dickinson, USA), with an air-cooled argon ion laser, 15 mW, 488 nm. Time-dependent assays were conducted over 1–3 min with a time resolution of 1 s. Recording of the fluorescence started 3 s after addition of sodium dithionite. For each measurement, the solution of dithionite was freshly prepared (1 M) in 0.1 M Tris (pH 10.0). The molar ratio between sodium dithionite and NBD was 1.6 × 10^8^. The geometric mean values of the fluorescence intensity distribution within every second were calculated. The particle solutions were diluted to a concentration of approximately 50 counts per second. The geometric mean of 50 consecutive counts was calculated. The highest and lowest calculated values of these geometric means were taken as the error limits of the method. Single particles were gated on the basis of their light scattering to separate them from the aggregates since there is a linear increase of forward scattering with the size of the aggregates. Measurements are sketched in Fig. 1 and have been extensively explained in^11^. The data were analysed using WinMDI 2.8 software.

### Fluorescence spectroscopy

Fluorescence spectroscopy measurements were performed using a Fluoromax 2 fluorescence spectrometer (Spec Industries., Inc., USA). The excitation wavelength was set at 467 nm for NBD.

## Results and discussion

As was shown in our previous works^11,12^ the diffusion of charged small molecules, in this case dithionite, in PEMs cannot be described in the framework of the classical Fickean diffusion. In the current work, we suggest a new approach to describe the subdiffusion of dithionite in the following PEM systems [PAH-PSS]_4_-[PAH^NBD^-PSS]_3_-PAH^NBD^, [PAH-PSS]_4_-[PAH^NBD^-PSS]_4_, [PAH-PSS]_4_-[PAH^NBD^-PSS]_7_-PAH^NBD^, [PAH-PSS]_4_-[PAH^NBD^-PSS]_8_. To trace the diffusion of the dithionite in the mentioned PEM systems the fluorescence decay of the label NBD was detected after the addition of the quencher dithionite by the methodology described in^11^ and sketched in Fig. 1. The quenching assay could be performed with just one labelled PAH layer but the assembly of several layers of PAH^NBD^ allows for a better theoretical modelling of the progressive diffusion of dithionite in the multilayers as we will show below. In Fig. 1 also are shown the changes in relative fluorescence with respect to time for the PEMs [PAH-PSS]_4_-[PAH^NBD^-PSS]_3_-PAH^NBD^, [PAH-PSS]_4_-[PAH^NBD^-PSS]_4_, [PAH-PSS]_4_-[PAH^NBD^-PSS]_7_-PAH^NBD^, [PAH-PSS]_4_-[PAH^NBD^-PSS]_8_ after adding dithionite are presented. These data were shown in^11^ too. The PEMs had either PAH or PSS as the outermost layer in order to evaluate the impact of the charge of the outermost layer on S_2_O_4_^2−^ diffusion.

From Fig. 1 it can be appreciated that all experimental curves display similar features. We observe a rapid quenching in the short time scale while the quenching process becomes largely retarded when we look over longer timescales. We can also observe from the decay curves that the number of assembled layers does not seem to affect dithionite diffusion for the considered 8 (7) and 16 (15) layers. This would mean that the surface charge of the PEM, either positive if the final layer is PAH, or negative if it is PSS, does not affect dithionite diffusion^11,12^.

The kinetics of the process of binding the quencher to the label can be divided into two stages—the diffusion stage of moving the quencher inside the polyelectrolyte film to the label and the kinetic stage, the actual binding of the quencher to the label. Experiments indicate that the kinetic stage in our systems is fast and the diffusion stage is slow^11,12^.

Based on this, it can be assumed that the process of the quencher binding with the label is limited by the diffusion of the quencher to the label in the polyelectrolyte film. The theoretical description of the binding kinetics of the quencher with the label will be carried out in the following model.

**Figure 2:**
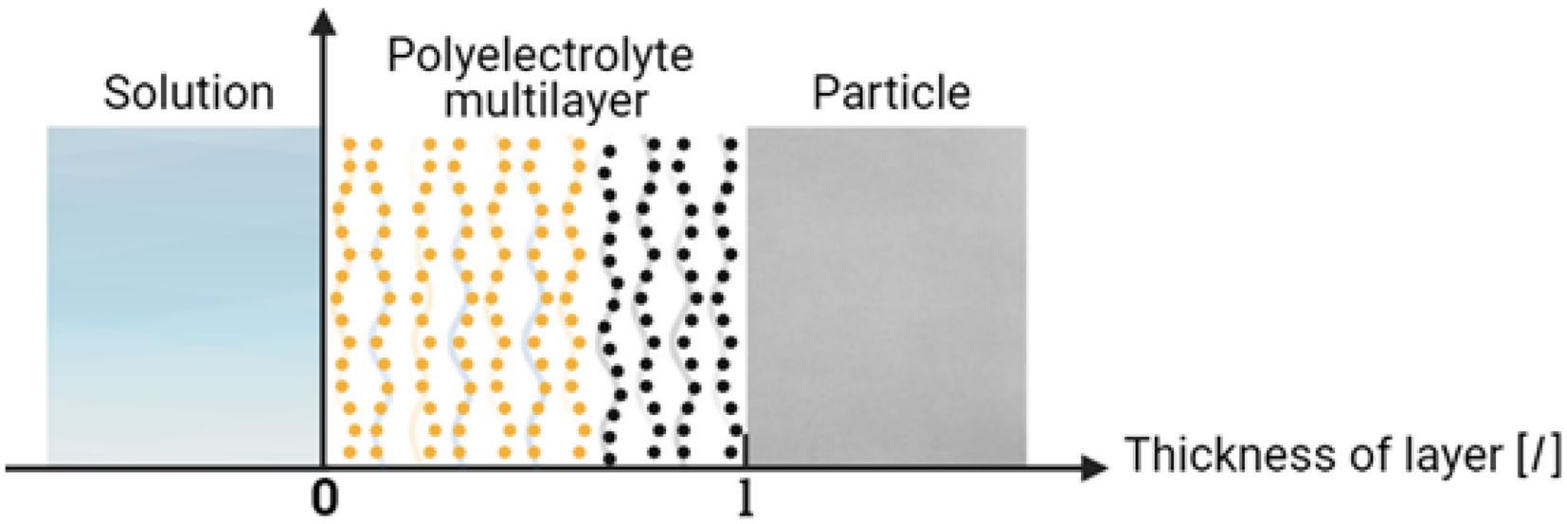
Displacement of quencher solution, polyelectrolyte film and substrate before the start of diffusion process. The thickness of polyelectrolyte film is *l*. The solution of quenchers (parallel lines) is located in region x ≤ 0, the labels, which are fixed on polyelectrolyte macromolecules (orange points) are located in region 0 < x < *l*, the substrate is located in region x ≥ *l*. On the second figure the profiles of initial concentrations of quenchers *C*_*Q*_ = *C*_*Q*_^*Eq*^ and labels *C*_*L*_ = *C*_*Lo*_ are presented. Fluorescence intensity corresponds to the shaded area under the concentration curve of labels.

Let us consider one-dimensional diffusion along the x-axis, which is perpendicular to the surface of the substrate on which the PEM with attached labels is located. The other surface of the polyelectrolyte film borders with the solution in which the quencher molecules are located. The origin of coordinates will be placed on the interface of the polyelectrolyte film-solution and the x axis will be directed toward the substrate on which the film is located (Fig. 2).

The thickness of the polyelectrolyte film is *l*. The concentration of the quenchers is kept constant in the solution, equal to *C*_*Q*_^*Eq*^. The concentration of quenchers at an arbitrary point of polyelectrolyte film depends on the x coordinate as well as on time *t*, i.e. we have *C*_*Q*_*(x,t)*. The boundary of the PEM with the substrate is impermeable for the quencher. At *t* = 0 the quencher’s concentration in the polyelectrolyte layers is 0, the initial concentration of fixed labels in polyelectrolyte film is *C*_*Lo*_. At an arbitrary point in time the concentration of the label is equal to *C*_*L*_*(x,t).* Initially all labels are intact, hence the fluorescence intensity is at a maximum. By the time quenchers penetrate into the polyelectrolyte film, gradually filling it, and when a quencher meets a label, a quasi-chemical reaction occurs, and the label is quenched. Since the binding process of a quencher with a label is fast, then the binding kinetics of a quencher with a label will be defined by the time of diffusive filling of the polyelectrolyte film. Correspondingly, the fluorescence intensity of the film will decrease as the quenchers reach the label layer.

First let us consider as we did in^11^, the quenching process when the quencher diffusion is described by the classical equation of diffusion. In the case of the classical description of diffusion the concentration profile of the quencher *C*_*Q*_*(x,t)* in a film *l* is defined by the following equation:

1 $$\frac{{\partial c_{Q} (x,t)}}{\partial t} = D_{Q} \frac{{\partial^{2} c_{Q} (x,t)}}{{\partial x^{2} }}$$

where *C*_*Q*_*(x,t)* is the number of quencher molecules in the polyelectrolyte film; *D*_*Q*_—the diffusion coefficient of quencher with *L*^*2*^*/ T* dimension. In order to solve Eq. (1) it is necessary to set the initial and boundary conditions. At t = 0 there is no quencher present in the vicinity of the polyelectrolyte layer, thus the initial condition for Eq. (1) will be the following:

2 $$c_{Q} (x,0) = 0$$

On the border with bulk solution the concentration of the quencher is constant and equal to *C*_*Q*_^*Eq*^, and the substrate is impermeable for the quencher. As such, the following boundary conditions will be defined:

3 $$\begin{gathered} c_{Q} (0,t) = c_{Q}^{Eq} \hfill \\ \left( {\frac{{\partial c_{Q} (x,t)}}{\partial x}} \right)_{x = l} = 0 \hfill \\ \end{gathered}$$

The solution of Eq. (1) with initial (2) and boundary (3) conditions are shown in the Supplementary Information. If the quencher concentration is known, then the kinetics of filling the film with quencher *θ(τ)* can be defined (*τ* is dimensionless time) according to the formula (9A) in supporting information. The quenching kinetics of label *R(τ)* is measured experimentally and is defined by *θ(τ)* by the following equation:

4 $$\frac{R(\tau )}{{R_{0} }} = 1 - \theta (\tau )$$

where R_0_ is the initial level of quenching. The analysis of the experimental data by the quenching kinetics of the label is described by the following dependence (5):

5 $$\begin{gathered} \frac{R(t)}{{R_{0} }} = \frac{8}{{\pi^{2} }}\sum\limits_{n = 0}^{\infty } {\frac{1}{{(2n + 1)^{2} }}\exp \left( { - \frac{{(2n + 1)^{2} \pi^{2} }}{4}\frac{t}{{t_{1} }}} \right)} \hfill \\ t_{1} = l^{2} /D \hfill \\ \end{gathered}$$

The fittings showing that in the framework of the classical description of the diffusion of a quencher in polyelectrolyte layers it is not possible to describe the experimental results are represented in the Supplementary Information (Fig. S1, supporting).

Since experimental quenching curves cannot be described with the assumption of a classical diffusion of dithionite in PEMs and diffusion is strongly retarded for dithionite in the multilayers, we considered here the possibility of subdiffusion of dithionite in the layers. Subdiffusion of dithionite could be occurring as the molecule is negatively charged and the PAH layers in the PEM have charged amine groups to which the quencher can attach electrostatically, thereby retarding its journey to the label and the quenching processes.

Let us describe the subdiffusion process of the quencher in a polyelectrolyte film using a diffusion equation with fractional time derivatives^13–16^. In this case Eq. (1) will have the following form:

6 $$\begin{gathered}_{0} D_{t}^{\alpha } c_{Q} (x,t) = D\frac{{\partial^{2} c_{Q} (x,t)}}{{\partial x^{2} }} \hfill \\ 0 < \alpha < 1 \hfill \\ \end{gathered}$$

where in the left part of Eq. (6) there is a fractional time derivative related to the quencher concentration^13–15^, and $D_{\alpha }$ is the diffusion coefficient of the quencher with the dimension $L^{2} /T^{\alpha }$. As in the initial moment of time in the layer there was no quencher and initial condition for Eq. (6) is the following:

7 $$\mathop {\lim }\limits_{t \to 0} \left( {_{0} D_{t}^{\alpha - 1} c_{Q} (x,t)} \right) = 0$$

The boundary conditions are the same as in (3). The procedure to calculate the label quenching kinetics is the same as for the case of classical diffusion (see the Supplementary Information). At first, the concentration of the quencher in the film is defined and then the kinetics of filling film with the quencher $\theta (\tau )$. Putting $\theta (\tau )$ in Eq. (4) the quenching kinetics of the label *R(τ)* will be defined. The analysis of the experimental data is convenient to perform according to the following dependence:

8 $$\begin{gathered} \frac{R(t)}{{R_{0} }} = \frac{8}{{\pi^{2} }}\sum\limits_{n = 0}^{\infty } {\frac{1}{{(2n + 1)^{2} }}E_{\alpha } \left( { - \frac{{(2n + 1)^{2} \pi^{2} }}{4}\left( {\frac{t}{{t_{1} }}} \right)^{\alpha } } \right)} \hfill \\ t_{1} = (l^{2} /D_{\alpha } )^{1/\alpha } \hfill \\ \end{gathered}$$

where $E_{\alpha } (z)$ is the Mittag–Leffler function.

**Figure 3:**
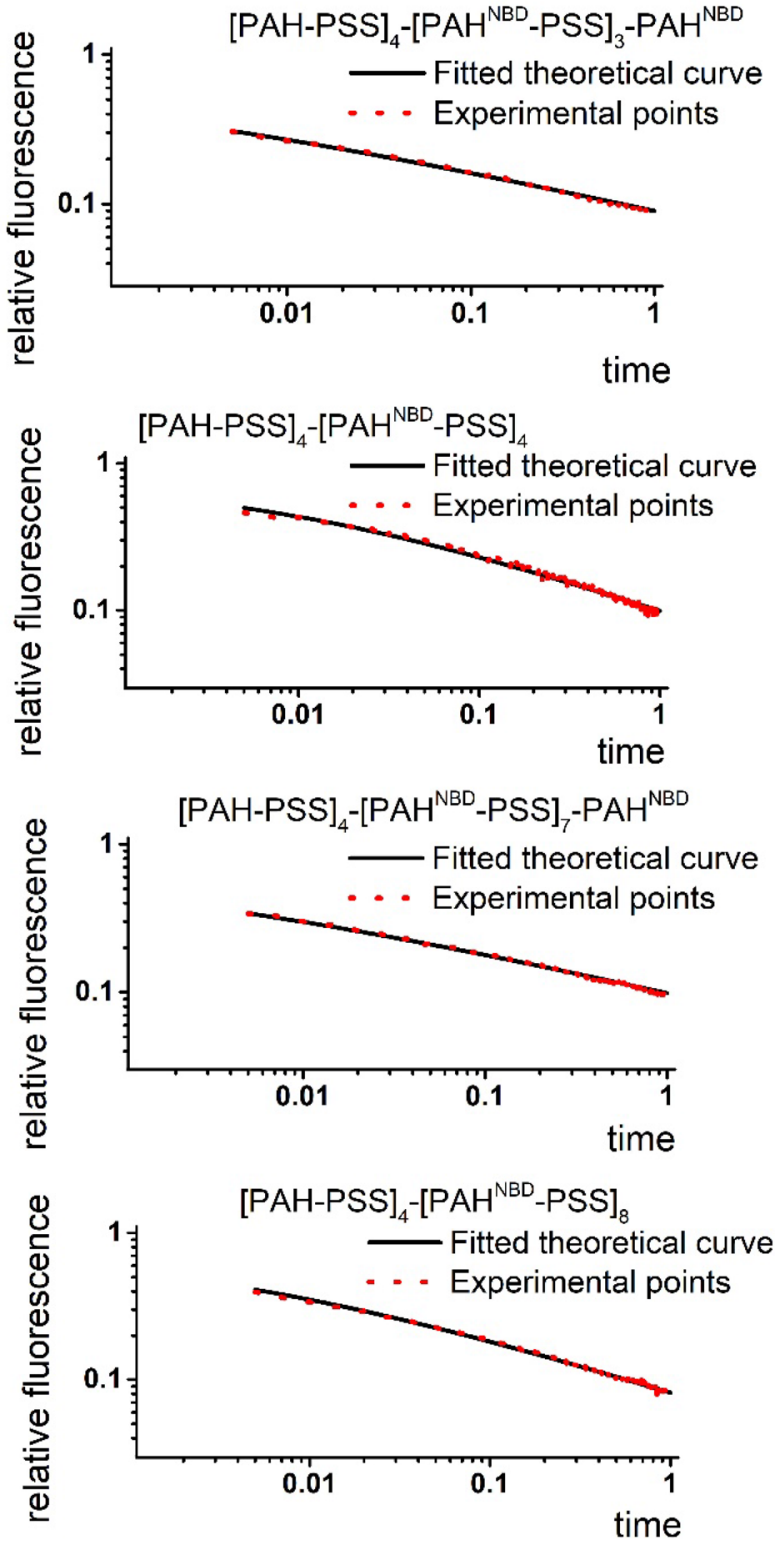
The fitting of experimental data with theoretical curve in log–log scale for [PAH-PSS]_4_-[PAH^NBD^-PSS]_3_-PAH^NBD^, [PAH-PSS]_4_-[PAH^NBD^-PSS]_4_, [PAH-PSS]_4_-[PAH^NBD^-PSS]_7_-PAH^NBD^, [PAH-PSS]_4_-[PAH^NBD^-PSS]_8_ and 20 mol/m^3^ dithionite.

From the fitting of experimental curvess with the theoretical curve obtained according to Eq. (8) it is possible to evaluate a numerical value of α and the coefficient of anomalous diffusion D_α_. The accuracy of the fitting to the experimental data is shown in Fig. 3.

The subdiffusion parameters for the mentioned polyelectrolyte multilayers are defined by the fitting and are presented in Table 1.

**Table 1:** Calculated values of α and Dα of the quencher in the layers.

The layers	α	D_α_, 10^–14^ cm^2^ s^−α^
[PAH-PSS]_4_-[PAH^NBD^-PSS]_3_-PAH^NBD^	0.29	2.87
[PAH-PSS]_4_-[PAH^NBD^-PSS]_4_	0.42	2.53
[PAH-PSS]_4_-[PAH^NBD^-PSS]_7_-PAH^NBD^	0.3	6.02
[PAH-PSS]_4_-[PAH^NBD^-PSS]_8_	0.39	7.29

Table 1 contains D_α_ values obtained from fitting for values of α between 0.29 and 0.42 and quencher concentrations equal to the bulk concentration. We obtain D_α_ values between 2.53 and 7.29 × 10^–14^ cm^2^ s^−α^. D_α_ does not change significantly for a similar number of layers if the last layer is positively or negatively charged, but increases in average 2.5 times when the thickness of the layers is increased two times. Similar behaviour for the diffusion parameters is shown in our previous work^11^ although it was shown that the diffusion process is described in the framework of a different approach where the diffusion coefficient depends on time D(t) = D_0_/t^1−α^. Nevertheless, the same variation of layers causes similar changes in the diffusion parameters in two different approaches of the description of slow diffusion.

**Figure 4:**
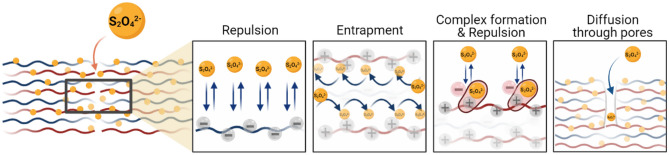
Schematics of the different situations experiencing S_2_O_4_^–2^ when diffusing through a PEM of PAH/PSS.

It should be noted that due to structural characteristics, the density of macromolecules in a polyelectrolyte film is high and this circumstance most probably makes the quencher diffusion process slow. The slow kinetics of label quenching can be explained within the framework of the classical diffusion equation for the quencher, taking into account the time dependence of the diffusion coefficient, as previously shown in^11,12^. However, dithionite may experience repulsive and attractive interactions with negatively and positively charged polyelectrolytes, respectively, while diffusing through the multilayer. We have sketched the different situations that dithionite may be facing during diffusion in the multilayers in Fig. 4. PEMs are not perfectly layered structures. There is interdigitation among subsequent layers and there is free space between layers and among polyelectrolyte chains in a layer. Diffusion of dithionite must take place through these free spaces in the multilayer, not occupied by polyelectrolytes and filled with solvent. We observe practically no differences in the quenching curves of dithionite when PEMs have a positive or a negative top layer, which indicates that the diffusion takes place without being affected by the repulsion or attraction from the top layer. Since dithionite has two negative charges, when it binds to the positive charges of PAH, it brings extra negative charges to the PEM, which may induce repulsive forces for the dithionite in solution, similar to when the top layer of the PEM is negatively charged. The increase in diffusion time with layer thickness can be explained as the layers become more compact as the number of assembled layers increases and there is less free space for dithionite diffusion between chains and layers. Once diffusing through free space in the top layers of the PEM dithionite may face either negatively charged PSS molecules or positively charged PAH molecules. The repulsive interaction with PSS will prevent dithionite from diffusing along this layer and force the dithionite to go through PAH layers on top and below. However, when the dithionite finds the amine groups of PAH it may form a complex to the positively charged amines, which act as traps, lingering dithionite from further diffusing. Once in contact with PAH dithionite may remain complexed with the amines jumping among them in a PAH layer before continuing diffusion. We observe an initial fast decay of fluorescence meaning that some dithionite molecules diffuse freely to the NBD labelled PAH layer, through voids in the layer filled with solvent. The fast decay is followed by a much slower quenching, which can be explained by the trapping of dithionite molecules in PAH layers and repulsion by PSS layers. Once dithionite interacts with PAH the positive charges of PAH are reversed by dithionite, which bears two negative charges per molecule creating additional repulsion for free dithionite molecules to go through the multilayers. In short, diffusion of dithionite will depend on the history of its displacement in the multilayers, if finds its way through voids in the multilayers till NBD or if gets trapped in the layers, in one case we have ordinary diffusion and in the other a slow one. Subdiffusion is a result of the particular organization of polymer chains in a multilayer, which can change with layer composition.

## Conclusions

In the current work it is shown that the slow diffusion of dithionite in PEMs can be described as a subdiffusion process. From the fitting of the quenching kinetics of labels in polyelectrolyte the parameters of subdiffusion—the diffusion coefficient and the order of fractional derivative are obtained.

From the obtained results it is possible to see the difference in the diffusion coefficient and order of fractional derivative for the positively and negatively charged PEMs. Namely, for the thinner films, e.g.: 7–8 layers, α depends on the charge of the outermost layers at the interface with the bulk solution and for positively charged PEMs it is approximately 0.3 and for negatively charged PEMs it is approximately 0.4. D_α_ does not change significantly for a similar number of layers. For thicker films, e.g.: 15–16 layers, α remains the same but the D_α_ increases approximately 2.5 times. In other words, for our films the parameter α is approximately 0.3 for positively charged PEMs and approximately 0.4 for negatively charged PEMs and does not appear to depend on the film thickness. The D_α_ parameter also appears not to depend on the charge of the outermost layer but increases for thick films.

The description of dithionite diffusion on the bases of the subdiffusion via fractional partial derivatives assumes that the diffusion process is non-Markovian, strongly affected by the history of the diffusion process. Subdiffusion of negatively charged dithionite in PEMs can be explained by the architecture of the PEMs, displaying voids with solvent, positively charged layers that act as traps, and negatively charged polymer layers that repeal dithionite, as well as by the presence of voids of solvent in the PEMs where the dithionite can diffuse freely.

## Supplementary Information

Supplementary Information.
