# Supplementary material for: A study of the subdiffusion of small molecules in charged polyelectrolyte multilayers
Source: Sci Rep. 2021 Nov 19;11:22585. doi: 10.1038/s41598-021-01935-7 (PMC8604972; doi:10.1038/s41598-021-01935-7)
Supplement: Supplementary file 1 — Supplementary Information. [file 41598_2021_1935_MOESM1_ESM.docx]

**A Study of the Subdiffusion of small Molecules in charged Polyelectrolyte Multilayers**

I. Vardanyan^1^, V. Arakelyan^1§^, Z. Navoyan^1^, Eleftheria Diamanti^2^, S.E. Moya^2§^, E. Donath^3^

^1^Yerevan State University, Department of Molecular Physics, 1 Al. Manoogian Str., Yerevan, 0025, Armenia

^2^Soft Matter Nanotechnology lab, CIC biomaGUNE, 20009 San Sebastian, Spain

^3^Institute of Medical and Biophysics, University of Leipzig, Haertelstrasse 16-18, Leipzig, Germany

§ Corresponding authors:

Valeri Arakelyan [v.arakelyan@ysu.am](mailto:v.arakelyan@ysu.am) Sergio Moya [smoya@cicbiomagune.es](mailto:smoya@cicbiomagune.es),

**Supplementary Information**

Classical diffusion.

Let us rewrite equation (1) initial (2) and boundary (3) conditions in dimensionless form. The following notations are introduced:

$$c_{1}=c_{Q}/c_{Q}^{Eq},\begin{matrix} & \xi=x/l,\begin{matrix} & \tau=t\cdot D_{Q}/ \end{matrix} \end{matrix}l^{2}$$

Then (1), (2) and (3) will take the following form:

$\frac{\partial c_{1}(\xi,\tau)}{\partial\tau}=\frac{\partial^{2}c_{1}(\xi,\tau)}{\partial\xi^{2}}$ (1A)

$c_{1}(\xi,0)=0$ (2A)

$\begin{matrix} c_{1}(0,\tau)=1 \\ \left(\frac{\partial c_{1}(\xi,\tau)}{\partial\xi}\right)_{\xi=1}=0 \end{matrix}$ (3A)

Equation (1A) is easily solved in a standard manner. As we are interested in the kinetics of filling film with the quencher it is expedient to use the Fourier method. The point of the method is that the solution is sought in the form of the product of two functions, one depending only on coordinates, and the other one only on time. The sequence of steps for solving equation (1) are the following. At first the problem is solved with homogenous boundary conditions. For that we switch from the function $c(\xi,\tau)$ to the function $c_{1}(\xi,\tau)=c(\xi,\tau)-1$. In this case the form of equation (1A) is unchanged, and the initial condition will be $c_{1}(\xi,0)=-1$. At the border $\xi=0$will have the condition $c_{1}(0,\tau)=0$ and on the border $\xi=1$ the condition will remain the same as in (3A). So, for the function $c_{1}(\xi,\tau)$ the equation (1A), conditions (2A) and (3A) will be in the following form:

$\frac{\partial c_{1}(\xi,\tau)}{\partial\tau}=\frac{\partial^{2}c_{1}(\xi,\tau)}{\partial\xi^{2}}$ (1AA)

$c_{1}(\xi,0)=-1$ (2AA)

$\begin{matrix} c_{1}(0,\tau)=0 \\ \left(\frac{\partial c_{1}(\xi,\tau)}{\partial\xi}\right)_{\xi=1}=0 \end{matrix}$ (3AA)

The solution of $c_{1}(\xi,\tau)$ is sought in the form of $c_{1}(\xi,\tau)=X(\xi)T(\tau)$ and its substitution in the partial differential equation (1A) leads to the solving of two regular equations of second order.

$\begin{matrix} X^{"}(\xi)=-\lambda^{2}X(\xi) \\ T^{"}(\tau)=-\lambda^{2}T(\tau) \end{matrix}$ (4A)

The solutions of equations (4A) are well known and have the following form:

$\begin{matrix} X(\xi)=C_{1}\cos(\lambda\xi)+C_{2}\sin(\lambda\xi) \\ T(\tau)=C_{3}\exp(-\lambda^{2}\tau) \end{matrix}$ (5A)

Where C_1_, C_2_ and C_3_ are arbitrary constants. Taking into account (5A) and redefining arbitrary constants $c_{1}(\xi,\tau)$ solution will have the following form:

$c_{1}(\xi,\tau)=C_{1}\cos(\lambda\xi)\exp(-\lambda^{2}\tau)+C_{2}\sin(\lambda\xi)\exp(-\lambda^{2}\tau)$ (6A)

Arbitrary constants$C_{1}$, $\lambda$ in (6A) are defined from boundary conditions (3AA) and are equal to $C_{1}=0$, $\lambda_{n}=(2n+1)\pi/2$. Afterwards the general solution $c_{1}(\xi,\tau)$ is represented in the form of sum of partial solutions:

$c(\xi,\tau)=\sum_{n=0}^{\infty} C_{n}\sin\left(\frac{(2n+1)\pi\xi}{2}\right)\exp\left(-\frac{(2n+1)^{2}\pi^{2}}{4}\tau\right)$ (7A)

where $C_{n}$ is reduced coefficient, which is defined from initial condition (2AA) and is equal to $C_{n}=-4/(\pi(2n+1))$. The final expression for $c(\xi,\tau)$ has the form of

$c(\xi,\tau)=1-\frac{4}{\pi}\sum_{n=0}^{\infty} \frac{1}{(2n+1)}\sin\left(\frac{(2n+1)\pi\xi}{2}\right)\exp\left(-\frac{(2n+1)^{2}\pi^{2}}{4}\tau\right)$ (8A)

The kinetics of film filling with quencher by time will change according to the following formula:

$\theta(\tau)=\int_{0}^{1} c(\xi,\tau)d\xi$ (9A)

Using the formula (8A) the label quenching kinetics is defined by the following formula

$\frac{R(\tau)}{R_{0}}=1-\theta(\tau)$ (10A)

where $R_{0}$ is the initial level of quenching. Putting the expression of $c(\xi,\tau)$ from (8A) into (9A) and then $\theta(\tau)$ into (10A) we will obtain the final expression for label quenching kinetics in the following form:

$\frac{R(\tau)}{R_{0}}=\frac{8}{\pi^{2}}\sum_{n=0}^{\infty} \frac{1}{(2n+1)^{2}}\exp\left(-\frac{(2n+1)^{2}\pi^{2}}{4}\tau\right)$ (11A)

For analysis of the experimental data, it is convenient to rewrite the expression (11A) as follows:

$\begin{matrix} \frac{R(\mathrm{t})}{R_{0}}=\frac{8}{\pi^{2}}\sum_{n=0}^{\infty} \frac{1}{(2n+1)^{2}}\exp\left(-\frac{(2n+1)^{2}\pi^{2}}{4}\frac{t}{t_{1}}\right) \\ t_{1}=l^{2}/\mathrm{D} \end{matrix}$ (12A)

Subdiffusion. Let us describe the diffusion process of the quencher using the diffusion equation with fractional time derivative [13-15]. Let us notice, that in many publications, where the fractional derivatives are used for describing real physical processes the fractional derivative is taken in the sense of Caputo [16]. We also will keep that approach. The sequence of the steps in case of solving the diffusion equations with fractional derivatives is the same as for classical diffusion equation. At first, let us rewrite the equation (5), initial (6) and boundary (3) conditions in dimensionless form

$\begin{matrix} _{0}D_{\tau}^{\alpha}c(\xi,\tau)=\frac{\partial^{2}c(\xi,\tau)}{\partial\xi^{2}} \\ 0<\alpha<1 \end{matrix}$ (13A)

$\lim_{\tau\to0} \left(_{0}D_{\tau}^{\alpha-1}c(\xi,\tau)\right)=0$ (14A)

$\left(\frac{\partial c(\xi,\tau)}{\partial\xi}\right)_{\xi=1}=0\begin{matrix} , & \end{matrix}c(0,\tau)=1$ (15A)

$$c=c_{Q}/c_{Q}^{Eq},\begin{matrix} & \xi=x/l,\begin{matrix} & \tau=t\cdot(D_{\alpha}/ \end{matrix} \end{matrix}l^{2})^{1/\alpha}$$

As in case of classical diffusion at first the problem is solved with homogenous boundary conditions. For that the function $c(\xi,\tau)$ is substituted with the function $c_{1}(\xi,\tau)=c(\xi,\tau)-1$. The equations (13A), conditions (14A) and (15A) will take the following form:

$\begin{matrix} _{0}D_{\tau}^{\alpha}c_{1}(\xi,\tau)=\frac{\partial^{2}c_{1}(\xi,\tau)}{\partial\xi^{2}} \\ 0<\alpha<1 \end{matrix}$ (13АА)

$\lim_{\tau\to0} \left(_{0}D_{\tau}^{\alpha-1}c_{1}(\xi,\tau)\right)=0$ (14АА)

$\left(\frac{\partial c_{1}(\xi,\tau)}{\partial\xi}\right)_{\xi=1}=0\begin{matrix} , & \end{matrix}c_{1}(0,\tau)=0$ (15АА)

The equation (13AA) is solved by Fourier method. The solution is sought in the form of $c_{1}(\xi,\tau)=X(\xi)T(\tau)$ and putting it into the (13AA) will give the system with two differential equations for $X(\xi)$ and $T(\tau)$. The equation for $X(\xi)$ coincide with corresponding equation for classical case and is solved in a standard way. The equation for $T(\tau)$ has the form

$\begin{matrix} _{0}D_{\tau}^{\alpha}T_{n}(\tau)=-\lambda_{n}^{2}T_{n}(\tau) \\ \lambda_{n}=\frac{(2n+1)\pi}{2} \end{matrix}$ (16A)

The solution of (16A) is well known and has the following form:

$T_{n}(\tau)=C_{n}E_{\alpha}(-\lambda_{n}^{2}\tau^{\alpha})$ (17A)

where $E_{\alpha}(z)$ is the Mittag-Leffler function. Then, as in classical case, is obtained general solution for $c_{1}(\xi,\tau)$ and Fourier series coefficients are defined and final solution is represented as follows:

$c(\xi,\tau)=1-\frac{4}{\pi}\sum_{n=0}^{\infty} \frac{1}{(2n+1)}\sin\left(\frac{(2n+1)\pi\xi}{2}\right)E_{\alpha}\left(-\frac{(2n+1)^{2}\pi^{2}}{4}\tau^{\alpha}\right)$ (18A)

Putting $c(\xi,\tau)$ from 18(A) into the (9A) and then the obtained $\theta(\tau)$ into the (10A) we will get the final expression for label quenching kinetics when the diffusion of a quencher in polyelectrolyte film is described by fractional derivative by time

$\frac{R(\mathrm{t})}{R_{0}}=\frac{8}{\pi^{2}}\sum_{n=0}^{\infty} \frac{1}{(2n+1)^{2}}E_{\alpha}\left(-\frac{(2n+1)^{2}\pi^{2}}{4}\tau^{\alpha}\right)$ (19A)

For analysis of experimental data, it is convenient to rewrite the expression in the following way:

$\begin{matrix} \frac{R(\mathrm{t})}{R_{0}}=\frac{8}{\pi^{2}}\sum_{n=0}^{\infty} \frac{1}{(2n+1)^{2}}E_{\alpha}\left(-\frac{(2n+1)^{2}\pi^{2}}{4}\left(\frac{t}{t_{1}}\right)^{\alpha}\right) \\ t_{1}=(l^{2}/D_{\alpha})^{1/\alpha} \end{matrix}$ (20A)

Let us notice that as $E_{1}(z)=exp(z)$ then as it was expected if $\alpha=1$ the expressions (20A), (19A) and (18A) will exactly become to the corresponding expressions for the case of classical diffusion (12A), (11A) and (8A).

The theoretical curves of label quenching kinetics in the case of normal diffusion of the quencher in the polyelectrolyte layer, which is described by classical diffusion equation (1), are presented in Figure 1 (corresponding curves, where α=1 is mentioned). Experimental points in Figure 1describing the label quenching kinetics have a characteristic profile – over short timescales the quenching is seen to be rapid, while over longer timescales the quenching process is strongly retarded. The character of theoretical curves in Figure 1A (for the cases where α=1) shows that in the framework of the classical description of the diffusion of a quencher in polyelectrolyte layers it is not possible to describe the experimental results, and diffusion can be considered atypical [11, 12].


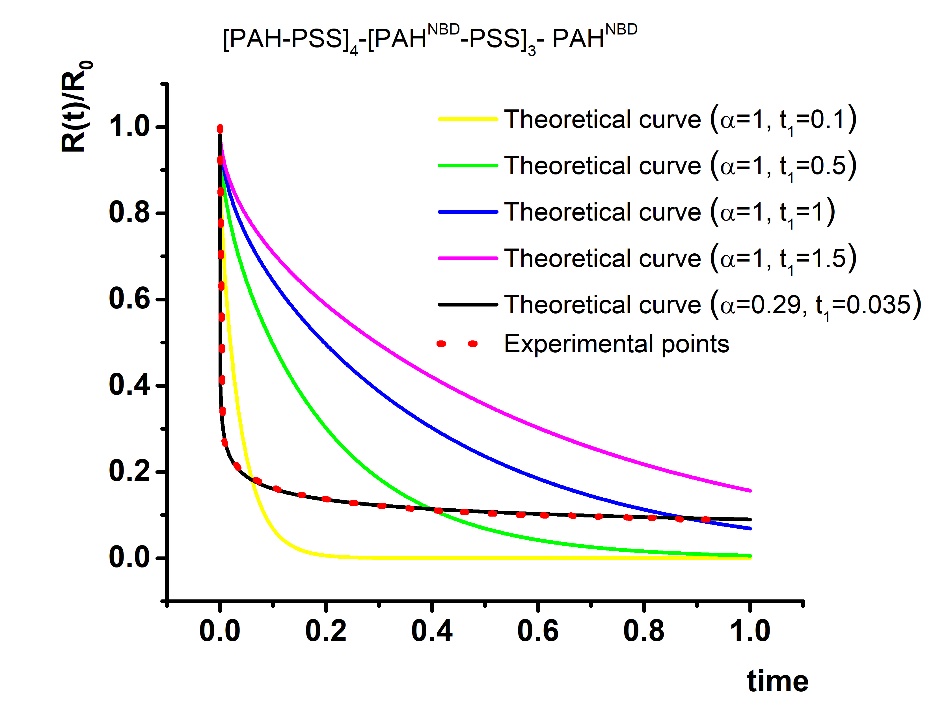


Figure S1. The dependence of label quenching kinetics on dimensionless time. The classical diffusion curves (with α=1) are obtained according to formula (12A). The curve with α=0.29 corresponds to the subdiffusion of the quencher in polyelectrolyte film and is obtained according to formula (20A).
